# Books and Magazines

**Published:** 1898-11

**Authors:** 


### Books and Magazines.

---

AMERICANS will gain a new estimate of the progress which the United States is making in the appreciation and the cultivation of art from reading Mr. William Sharp's description of The Art Treasures of America, reprinted from The Nineteenth Century in *The Living Age* for October 29.

---

EMERSON'S SON AS AN AUTHOR.—Ralph Waldo Emerson's son, Dr. Edward Emerson, himself a boy when Louisa Alcott was a girl in Concord, has written an article on "When Louisa Alcott was a Girl," which the *Ladies' Home Journal* is about to publish. Dr. Emerson gives a new view of the author of "Little Men"—as a mimic, and as the central figure of every dance and merrymaking in old Concord.

---

THE LEADING FEATURES of the *American Monthly Review of Reviews* for November are: the editorial comment on the State and Congressional campaigns (illustrated); an illustrated account of the work of the "Y. M. C. A." in connection with the army and navy during the war with Spain, by Albert Shaw; an article on "The Newspaper Correspondents in the War," with numerous portraits; Mr. Creelman's own story of his Santiago adventures; "Ouida's" "Impeachment of Modern Italy," with Signor Vecchia's reply; "The Nicaragua Canal in the Light of Present Politics," by Prof. L. M. Keasbey; and "The Nicaragua Canal and Our Commercial Interests," by Dr. Emory R. Johnson.

---

"HISTORY OF THE SPANISH-AMERICAN WAR," BY HENRY WATTERSON.—The above is the title of a superbly illustrated, richly bound volume issued by The Werner Company, Akron, Ohio. It is the only authentic history of the Spanish-American War that has so far come to our notice. All the others have, in the main, been merely re-vamped histories of the Cuban War, with some illustrations and a few chapters about the Spanish-American War. Of course, anything written by Henry Watterson would be readable. He, above all other men in America, is fitted by training and experience to write a history of this war, which has brought world-wide renown and glory to our arms. Every line of the book breathes an enthusiastic spirit of patriotism that is exhilarating and inspiring.

The work contains over 650 pages, a large number of full-page half-tones, together with many rich double-page illustrations in ten colors. It is sold by subscription, and will undoubtedly prove a money-maker to every intelligent salesman.

